# A CDK-4EBP1 signaling axis drives HSV-1 replication and underscores a druggable pathway for potent antiviral intervention

**DOI:** 10.1128/mbio.03741-25

**Published:** 2026-01-23

**Authors:** Krishnaraju Madavaraju, Tejabhiram Yadavalli, Sudhanshu Kumar Singh, Chandrashekhar D. Patil, Hemant Borase, Deepak Shukla

**Affiliations:** 1 Department of Ophthalmology and Visual Science, University of Illinois Chicago 14681, Chicago, Illinois, USA; 2 Department of Microbiology and Immunology, College of Medicine, University of Illinois Chicago 12247, Chicago, Illinois, USA; Tsinghua University, Beijing, China

**Keywords:** herpes simplex virus-1 (HSV-1), 4EBP1, GW8510, CDK, BX795

## Abstract

Herpes simplex virus type 1 (HSV-1) poses a persistent public health challenge, particularly due to the emergence of drug-resistant strains and the limited efficacy of current monotherapies. Through an unbiased multi-omic approach, we identify a previously lesser-known viral strategy in which HSV-1 hijacks cyclin-dependent kinase (CDK) signaling to disrupt host cell cycle and translational control, specifically via the eukaryotic translation initiation factor 4E-binding protein 1 (4EBP1). Targeted knockdown of CDKs confirmed their critical role in mediating 4EBP1 dephosphorylation during infection. Mechanistic evaluation of BX795, a previously known modulator of the 4EBP1 pathway, revealed an alternative route of translational repression mediated through CDKs. To further support this conclusion, we demonstrated that a distinct small-molecule CDK inhibitor, GW8510, exhibits potent antiviral activity against HSV-1 and functions as a true mechanistic analog of BX795. Together, these findings uncover a previously unrecognized CDK-4EBP1 regulatory axis exploited by HSV-1 and identify GW8510 as a promising candidate for host-directed antiviral intervention.

**IMPORTANCE:**

Herpes simplex virus type 1 remains a major clinical burden, and resistance to existing therapies underscores the need for alternative strategies. This study reveals a mechanism by which HSV-1 regulates host cell cycle and translation control through cyclin-dependent kinase signaling and the 4E-binding protein 1 pathway. By revealing that pharmacological inhibition of this pathway suppresses viral replication, we identify a host-directed therapeutic approach that circumvents challenges associated with viral resistance to the current drugs. The demonstration of potent antiviral activity by GW8510, a small-molecule cyclin-dependent kinase inhibitor, establishes a promising foundation for translational development and highlights the potential of targeting host regulatory networks to combat viral infection.

## INTRODUCTION

Herpes simplex viruses (HSV) are among the most ubiquitous pathogens known to humankind (1, 2). These viruses cause a variety of pathologies, ranging from opportunistic infections in immunocompromised patients to severe central nervous system complications in neonates. Most importantly, HSV can infect seemingly most common cell and tissue types in a variety of species, ranging from zebrafish to humans (3). This efficiency in infection can be attributed to its ability to exploit a variety of host factors to its own advantage and its ability to cause recurrent infections (4).

One of the most disastrous consequences of HSV, specifically type 1, infection occurs when the virus infects the eye (5–9). While all ocular tissues can be involved, involvement of the cornea is more common and vision-threatening (10, 11). Primary infections are typically observed in children or adolescents and are associated with vesicular dermatitis, follicular blepharoconjunctivitis, superficial punctate keratitis (SPK), or dendritic ulcers, often accompanied by preauricular lymphadenopathy (12). Recurrent infections are a major problem due to their potential to cause corneal scarring as sequelae. The involvement of the cornea can be epithelial, stromal, or endothelial (13). While the diversity in pathogenesis is predominantly dependent on the patient’s immune status, differences in viral modulation of host factor expression significantly contribute to the prognosis of ocular disease (13–16). Therapies in the form of nucleoside analogs are highly effective in controlling these viral infections, but HSV can develop resistance to these drugs (17–20). In this regard, there is an urgent need to develop alternative therapies that function through different mechanisms of action and enable effective control of the virus. Hence, it is essential to recognize how the virus modulates the host cell machinery, especially in the corneal tissue. Previous reports have provided significant insight into how the virus modulates the host transcriptomic and proteomic profiles in multiple tissue types, including fibroblasts, human skin xenografts, central and peripheral neuronal tissues, and various cell types (21–29). However, HSV-1 infects, replicates, and recurs predominantly in the corneal epithelium and stroma; therefore, it is essential to investigate its modulatory behavior in these cell types if suitable therapies are to be developed.

In this study, we used primary human corneal epithelial cells infected with HSV-1 to perform unbiased transcriptomic and proteomic analyzes, assessing differentially modulated pathways in the corneal tissue. Our studies indicate that the cyclin-dependent kinase (CDK)-mediated cell cycle pathways are the most crucial for viral replication in the cell. Utilizing multi-omic and molecular dynamics simulation studies, we demonstrate that BX795 and, more importantly, its functional analog GW8510 function through these virus-exploited pathways, exhibiting excellent antiviral efficacy.

## RESULTS

### HSV-1 infection reprograms corneal epithelial cell transcriptome and proteome toward cell cycle activation

**Fig 1:**
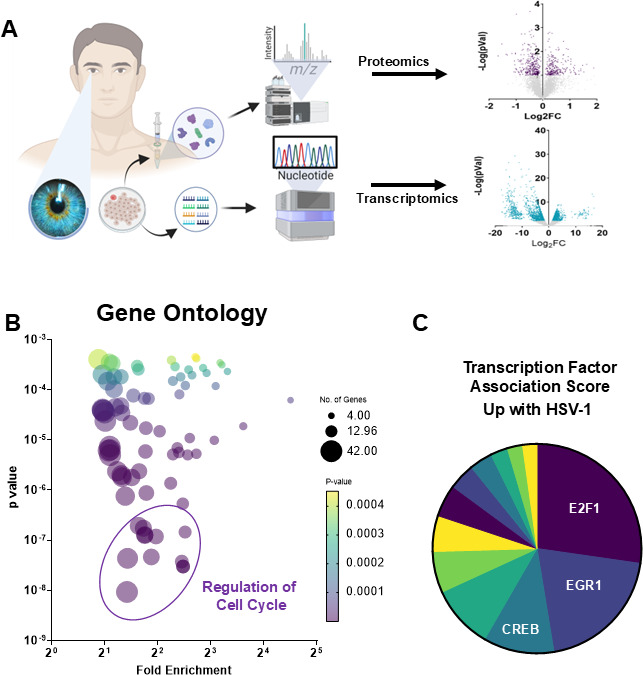
HSV-1 infection activates host cell cycle pathways via E2F1. (**A**) Primary human corneal epithelial cells were either left uninfected or infected with HSV-1, then processed in parallel for transcriptomic (RNA) and proteomic analysis (*n* = 3 biological replicates). Combined multi-omic analysis identified numerous host genes and proteins significantly up- or downregulated by HSV-1 infection. (**B**) GO enrichment analysis of the differentially expressed host genes/proteins shows that the cell cycle is the most significantly affected biological process in HSV-1-infected cells. (**C**) PASTAA transcription factor affinity analysis of upregulated host gene sets reveals E2F1 as the top activated transcription factor during HSV-1 infection.

Human cadaver corneas were processed to isolate primary human corneal epithelial cells (HCEs). These cells were either left non-infected or infected with the HSV-1 KOS strain at a 0.1 MOI for 24 h. Experiments were performed in triplicate and were simultaneously processed for proteomic and transcriptomic analysis (Fig. 1A). Results from both omic analyzes revealed a significant number of differentially regulated genes. Both data sets were combined to generate a single list of top upregulated and downregulated genes. Gene ontology (GO) term analysis of differentially expressed genes revealed regulation of the cell cycle as the most significant process during HSV-1 infection (Fig. 1B). Furthermore, predicting associated transcription factors from annotated affinities (PASTAA) analysis (30) showed that among the significantly upregulated gene sets identified, E2F1 was the most active transcription factor during HSV-1 infection (Fig. 1C). The E2F family of transcription factors plays a crucial role in regulating the host cell cycle, DNA replication, and the DNA damage response, which validates our GO term analysis that HSV-1 closely regulates the host cell cycle (31, 32).

#### HSV-1 replication in human cornea is facilitated by CDK-mediated E2F transcription

**Fig 2:**
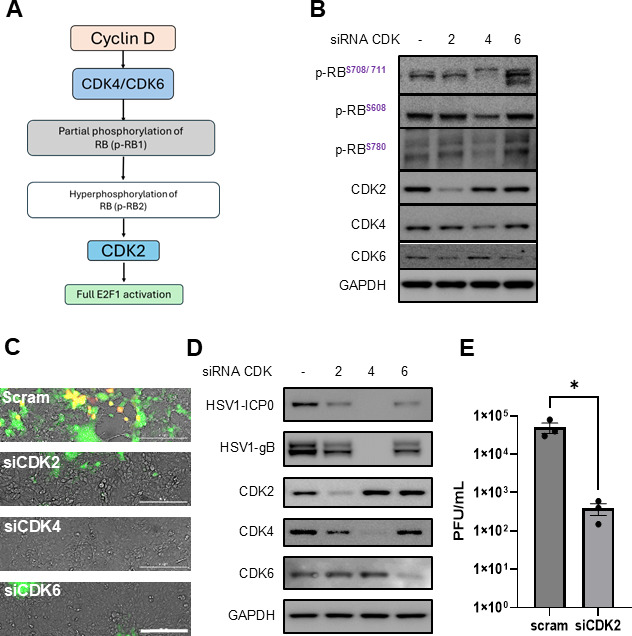
CDK-mediated E2F transcription facilitates HSV-1 replication. (**A**) Schematic showing the CDK-mediated E2F1 activation. (**B**) Immunoblot analysis of RB phosphorylation in uninfected cells following siRNA-mediated knockdown of CDK2, CDK4, or CDK6 (48 h knockdown). Phosphorylation of RB at Ser^708/711^ and Ser^608^ is markedly reduced by CDK4 knockdown compared with control, indicating CDK4’s role in RB-E2F regulation (representative blot from three independent experiments). (**C**) HSV-1 infection is significantly inhibited in cells deficient in CDK2 or CDK4. Viral replication was assessed 24 h post-infection using the HSV-1 GFP reporter virus, which showed a pronounced decrease in fluorescence intensity in CDK2- or CDK4 knockdown cells compared with the control. (**D**) Knockdown of CDK2 or CDK4 also reduces HSV-1 viral protein ICP0 and gB expression in infected cells and the production of HSV-1 virus, as demonstrated by Western blot analysis and (**E**) plaque assay. The data represents three independent experiments. Statistical significance was evaluated by Student’s *t*-test *P < 0.05.

As E2F1 is regulated through the phosphorylation of retinoblastoma protein (RB) by CDKs (Fig. 2A) (31, 32), we attempted to knock down CDK2, 4, and 6, which have known roles in HSV-1 infection and RB phosphorylation. In non-infected HCE cells, we found that RB phosphorylation at serine 708, 711, and 608 was most downregulated by the transient knockdown of CDK4 (Fig. 2B). On the other hand, in infected cells, all three CDK2 and CDK4 knockdowns showed promising antiviral activity, as indicated by the decrease in HSV-1-mediated fluorescence (Fig. 2C) and viral proteins, including infected cell protein (ICP0) and glycoprotein B (gB) (Fig. 2D).

While the inhibition of RB phosphorylation explained the loss of HSV-1 infection by CDK4 knockdown, the process through which CDK2 knockdown worked remained elusive for HCE) cells. Previous reports have hinted that the knockdown of CDK2 inhibits the initiation of viral replication (33–35). Our transient knockdown of CDK2 in HCE cells using siRNA resulted in a significant suppression of HSV-1 replication, as evidenced by the loss of HSV-1 GFP production and reduced plaque counts (Fig. 2C and D).

#### CDK2 inhibition disrupts protein translation

**Fig 3:**
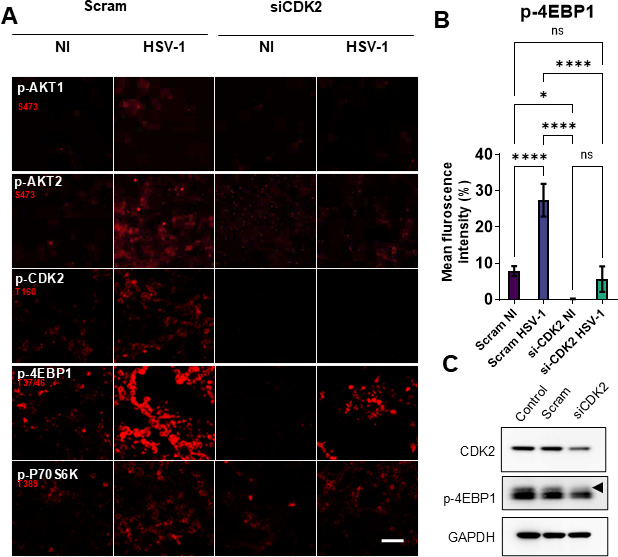
CDK2 inhibition disrupts 4EBP1 phosphorylation. (**A**) siCDK2 knockdown of human corneal epithelial cells, whether uninfected or HSV-1-infected, shows a marked reduction in levels of phospho-4EBP1 compared with controls. (**B**) Quantification of three independent imaging experiments showing relative fluorescence units of p-4EBP1. (**C**) Immunoblot demonstrating that CDK2 knockdown reduces 4EBP1 hyperphosphorylation in non-infected cells. All quantitative data are from *n* = 3 independent experiments (mean ± SD). siCDK2 was used at a concentration of 10 nM. Statistical significance was evaluated by one-way ANOVA with post hoc tests (for multiple groups); significance is indicated as ns-nonsignificant, *P < 0.05 or ****P < 0.0001.

Given the role of CDK2 in translation, we aimed to check the response to CDK2 inhibition on the proteins involved in this process. To our surprise, we found that CDK2 inhibition resulted in the loss of hyper-phosphorylation of eukaryotic initiation factor 4E binding protein 1 (4EBP1), including those of non-infected cells (Fig. 3A through C). Furthermore, we developed a large-scale confocal imaging assay using different primary antibodies in the 4EBP1 pathway (protein kinase B/AKT and P70S6 kinase) (Fig. 3A). The analysis of the imaging experiment indicated that transient knockdown of CDK2 using siRNA resulted in the loss of 4EBP1 and p70S6 kinase phosphorylation in non-infected cells, without inhibiting Akt (Fig. 3C). While the inhibition of phosphorylated Akt in infected cells during siCDK2 treatment can be attributed to the suppression of the virus, the results in non-infected cells are of interest and show that CDK2 inhibition indeed suppresses 4EBP1 phosphorylation. This surprising discovery suggests that HSV-1, through CDK2, can control the host cell translational machinery independently of protein kinase B (Akt) or the molecular target of rapamycin complex (mTORC1), which regulates the canonical pathway (36–39).

#### BX795 arrests the cell cycle through the inhibition of CDK2

**Fig 4:**
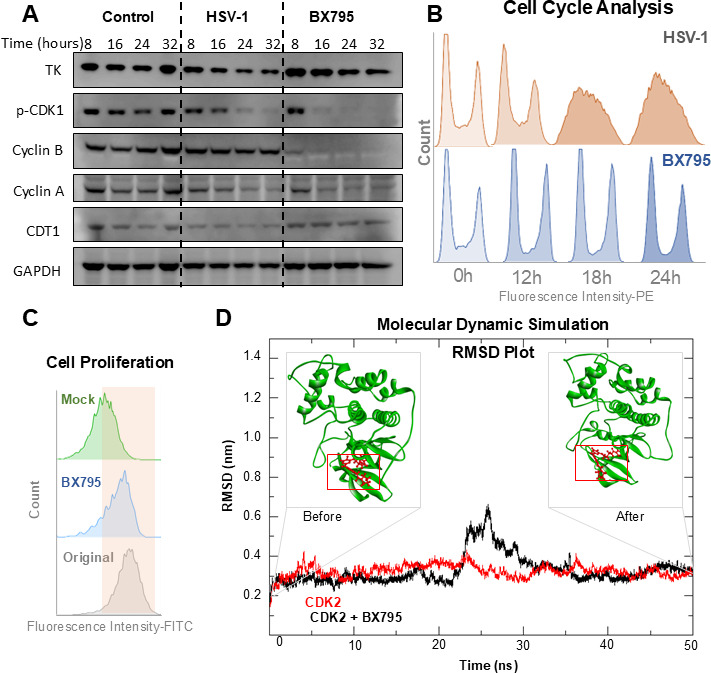
CDK2 knockdown mimics the effects of BX795 on translational control and cell cycle arrest. (**A**) Western blot of cell cycle regulators showing that treatment with BX795 (10 µM, 16 h) significantly reduces the expression of phosphorylated CDK1 and cyclin B in human corneal epithelial cells relative to DMSO-treated controls. (**B**) Cell cycle analysis (PI-stained DNA measured through flow cytometry) indicates that BX795 treatment (10 µM, 24 h) causes an arrest at the G₁/S boundary, in contrast to HSV-1 infection. (**C**) A proliferation assay (72 h, CellTrace/CytoPainter dye dilution) shows that only ~10% of BX795-treated cells enter the proliferation zone, whereas the majority of untreated cells replicate during the same period. (**D**) *In silico* modeling of BX795 binding to CDK2 confirms a stable interaction. Molecular docking followed by a 50 ns molecular dynamics simulation shows that BX795 remains stably bound in the CDK2 ATP-binding pocket, with minimal change in root-mean-square deviation over time (**D**).

We have previously demonstrated that BX795 exhibits potent antiviral activity against HSV-1 in corneal epithelial cells at a concentration of 10 µM (40–43). BX795, an aminopyrimidine molecule, was originally developed as part of a screen that demonstrated potent PDK1 activity for treating cancer cells (44). Later, in a kinase activity inhibition screen, it was shown to have the highest activity against TANK binding kinase-1 (TBK1) at concentrations of 10 and 100 nM (45, 46). Our previous studies hypothesized that BX795 treatment results in the loss of 4EBP1 hyperphosphorylation at the Thr37/46 site by inhibiting Akt phosphorylation (40). With the knowledge that CDK2 inhibition can also result in the loss of 4EBP1 phosphorylation, we hypothesized that BX795 may utilize this pathway instead of the canonical Akt pathway. To test this hypothesis, we performed Western blot analysis of key cell cycle-related proteins, which showed a significant loss of phospho-CDK1 and cyclin B within 16 h of BX795 treatment in HCE cells (Fig. 4A). Furthermore, mRNA transcript analysis through RT-qPCR of cells treated with BX795 showed a significant loss of CDK1 and cyclin B transcripts by the 8-h time point (Fig. S2A). These results indicated that BX795 inhibited CDK2 upon treatment. Furthermore, we performed cell cycle and cell proliferation analysis on HCE cells. It is well-known that HSV-1 modulates the cell cycle, ultimately causing the cell to enter a G1-S block (43, 44), which enables the host cell to produce large amounts of viral DNA (Fig. 4B). The treatment of HCE cells with BX795, however, completely depleted cell populations in their S-phase, suggesting a block in the G1/S and S/G2 transition. In line with these results, BX795 also inhibited cell proliferation over a 72-hour period, with a minimal number of cells replicating during this time. Cell proliferation was assessed using the CytoPainter kit, followed by flow cytometric analysis of the cells at the designated time point. The reduction in fluorescence intensity was indicative of replicating cells, and BX795 treatment resulted in approximately only 10% of cells in the proliferation zone (Fig. 4C). We performed *in silico* analysis to fit BX795 to CDK2, further affirming our results and understanding the potential binding site. Molecular dynamics simulation studies showed that the stability of BX795 in the active region of CDK2 did not significantly diverge from its original position up to 50 ns (Fig. 4D) and that the structure was stable without a decrease in hydrogen bonds, change in radius of gyration, and solvent accessible regions (Fig. S2B through E).

#### Pan-CDK inhibition reverses the cell cycle modulatory role of HSV-1

**Fig 5:**
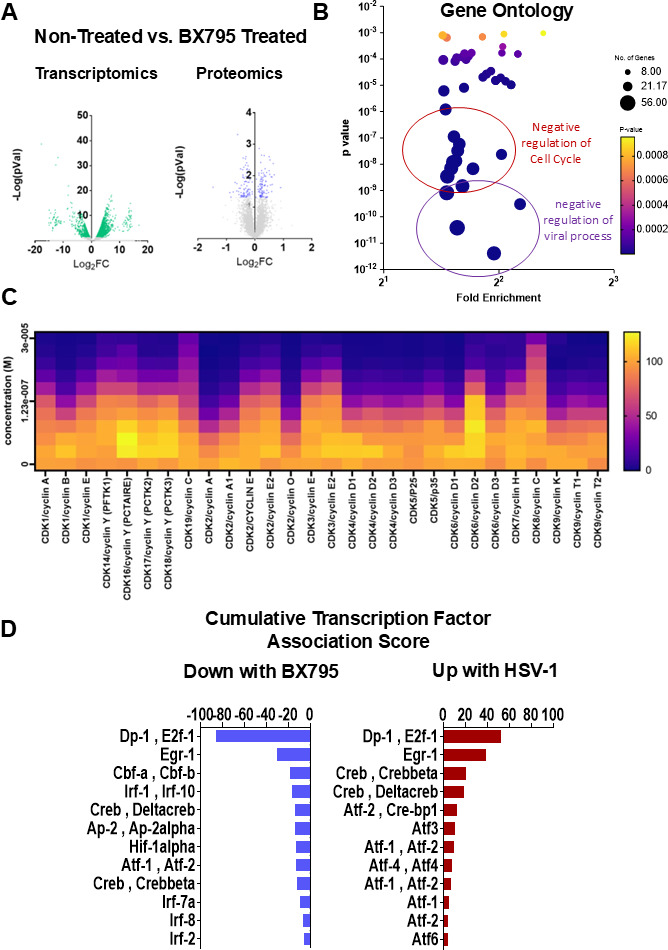
BX795 induces an antiviral state by acting as a pan-CDK inhibitor and modulating cell cycle genes. (**A**) Volcano plots summarizing transcriptomic and proteomic changes in primary human corneal epithelial cells treated with BX795 (10 µM for 24 h) versus vehicle control (*P* < 0.05 cutoff; *n* = 3 biological replicates for each omics analysis). (**B**) GO analysis of the overlapping set of significantly regulated genes/proteins shows negative regulations of cell cycle and viral process. Pathways in BX795-treated cells. (**C**) Kinase inhibition profile of BX795 against CDKs (Reaction Biology, 30 µM BX795 with threefold serial dilutions). The heat map indicates the percent inhibition of CDK activity. (**D**) Side-by-side comparison of PASTAA analysis of BX795-treated vs control and HSV-1 infected vs non-infected transcriptomes.

In addition to molecular dynamics simulation, our protein docking studies revealed excellent binding scores for BX795 with multiple CDKs, including CDK4 and CDK6 (Fig. S3A). To further expand our understanding of the critical cellular mechanisms activated or disrupted by BX795, we performed an unbiased multi-omics analysis on primary HCE cells treated with either BX795 or a DMSO vehicle control. Both poly-A mRNA transcriptomic and whole cell proteomic analyses were performed on these cells. The results from our data indicated approximately 3,500 significantly differentially expressed genes and 600 proteins between non-treated and BX795-treated cells, as shown in the volcano plots (Fig. 5A; Fig. S3B). Based on GO term analysis of differentially expressed genes and proteins between non-treated and BX795-treated cells, we observed that pathways representing “defense against virus,” “kinase regulatory activity,” and “DNA/RNA metabolic processes” were upregulated with BX795 treatment (Fig. S3C and D). Next, rather than analyzing the transcriptomic and proteomic data sets independently, we identified gene IDs that were consistently and significantly up- or downregulated in both data sets and then performed GO analysis on this combined set. These results interestingly showed the role of BX795 in the significant modulation of cell cycle-associated pathways (Fig. 5B). Combined, these results show that BX795 treatment at 10-µM concentration can significantly induce an antiviral state in corneal epithelial cells by modulating cell cycle-associated pathways. This result aligns with and validates earlier findings revealing a cell cycle- and cell proliferation-inhibitory role for BX795.

Previous studies have shown that BX795 at a concentration of 10 nM is a potent TBK1 inhibitor; however, a 10-fold increase to 100 nM already showed >90% inhibition of CDK2 in a different follow-up study (Fig. S4A) (47). Given that most of our antiviral observations were seen at a concentration of 10 µM, we performed a pan-CDK screen using a kinase inhibition activity assay (Reaction Biology) for BX795 starting at a concentration of 30 µM, followed by threefold dilutions (Fig. 5C). Our results indicate that BX795 is an inhibitor of most CDKs, with the highest activity against CDK2/cyclin A, CDK2/cyclin O, CDK4/cyclin D1, and CDK6/cyclin D1.

To our surprise, PASTAA analysis of DMSO or BX795-treated transcriptomic data sets revealed that indeed E2F1, which was the most exploited transcription factor during HSV-1 infection, was also the most inhibited during BX795 treatment (Fig. 3D). Together, these results indicate that BX795 is a pan-CDK inhibitor with cell cycle regulatory activities, exhibiting potent antiviral activity through the inhibition of CDK4 and controlling protein translation by inhibiting CDK2.

### Connectivity map analysis identifies GW8510 as a functional analog of BX795

**Fig 6:**
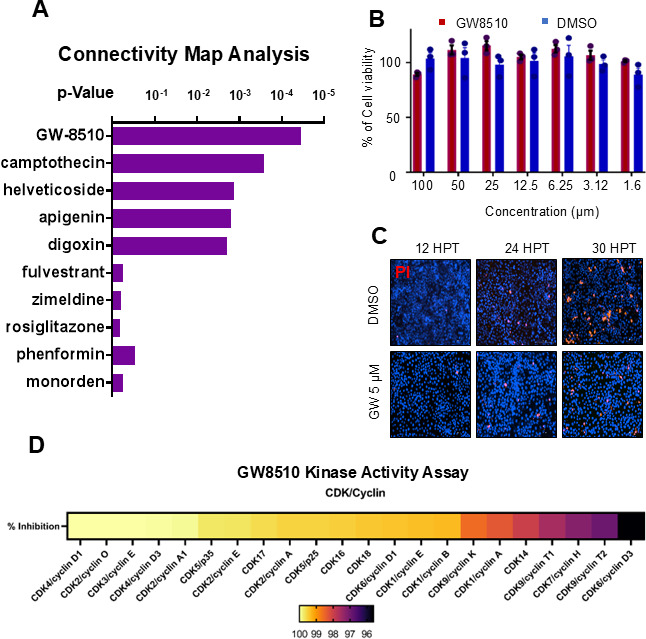
GW8510, a CDK inhibitor identified as a BX795 analog. (**A**) Connectivity map analysis of BX795 transcriptomic data reveals multiple drugs, including GW8510, as most like BX795. (**B**) Cell viability and (**C**) cytotoxicity assessment of GW8510 at higher or working concentrations. HCE cells treated with 5 μM of GW8510 for 24 h show no significant loss of viability compared with untreated cells (viability ≥ 95%, *P* > 0.05), indicating minimal host cell toxicity. (**D**) Kinase inhibition profile of GW8510 (5 μM) across a panel of cyclin–CDK complexes. GW8510 exhibits the strongest inhibition of CDK4/cyclin D1 and CDK2/cyclin A (greater than 90% inhibition of activity), with appreciable inhibition of other CDKs to lesser degrees. These results identify GW8510 as a functional analog of BX795 that potently inhibits CDK4 and CDK2, explaining its ability to block HSV-1 replication at lower, non-toxic concentrations.

Finally, we performed a connectivity map (CMAP) analysis (48) using the differentially expressed gene sets from our transcriptomic and proteomic data. CMAP is a novel tool that can provide perturbational analysis of a disease model and accelerate therapeutic discovery based on the perturbagen’s genetic signature. CMAP analysis of non-infected HCEs revealed that BX795 treatment resulted in a significant cell cycle inhibition. More importantly, CMAP analysis generated a list of the most similar and dissimilar drugs based on the curated input. To our delight, our analysis showed that GW8510 (4-{[(7-oxo-6,7-dihydro-8H-[1,3] thiazolo [5,4-e]indol-8-ylidene)methyl]amino}-N-(2-pyridyl) benzenesulfonamide), a previously known CDK2 inhibitor (49), was a functional analog of BX795 (Fig. 6A). In this regard, our study not only provides a novel mechanism of action for BX795 but also reveals its functional analog, GW8510, which could have potentially beneficial effects against HSV-1 infections in the eye. GW8510 at molar concentrations lower than those of BX795 does not exhibit toxicity in HCEs (Fig. 6B and C). A kinase activity assay performed using GW8510 revealed that it had the highest inhibitory activity against CDK4 and CDK2 at a concentration of 5 µM (Fig. 6D).

### GW8510 suppresses HSV-1

**Fig 7:**
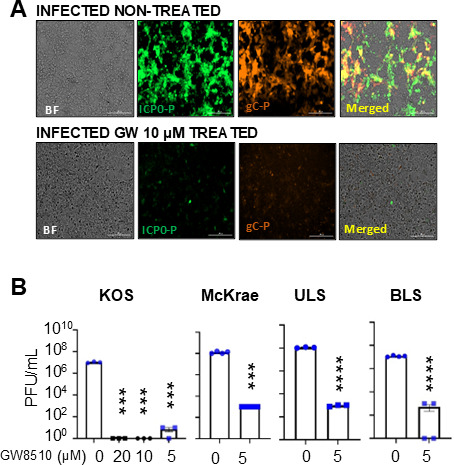
GW8510 effectively blocks different strains of HSV-1. (**A and B**) GW8510 exhibits potent antiviral activity against HSV-1 at 5 µM *in vitro*, against both laboratory and clinical HSV-1 strains, as measured by plaque assays or viral fluorescence. Data are representative of three experiments: statistical significance by one-way ANOVA, ***P < 0.001 or ****P < 0.0001.

Next, we aimed to determine whether the BX795 analog, GW8510, impairs HSV-1 infection. Our results supported the hypothesis. GW8510 significantly suppressed HSV-1 infection and disrupted key host-cell events required for productive viral replication and subsequent spread. At concentrations as low as 5 µM, the compound exhibited a strong antiviral effect against HSV-1 (Fig. 7A).

We next used a viral plaque assay to evaluate its effect on laboratory-adapted HSV-1 strains (KOS and McKrae). The drug clearly inhibited plaque formation by both strains (Fig. 7B). Because primary clinical isolates of HSV-1 often differ substantially in their biological behavior, we also tested two recently isolated clinical strains, BLS and ULS (50). To our satisfaction, GW8510 effectively blocked infection by both clinical isolates (Fig. 7B). This is an important result because clinical HSV-1 isolates maintain the genetic diversity, virulence attributes, innate immune antagonism, and drug-susceptibility profiles present in circulating viruses, including features linked to antiviral resistance, variable replication kinetics, and differential sensitivities to host-targeted therapeutics. The robust antiviral activity of GW8510 against multiple clinical isolates strongly supports the compound’s mechanism as a host-directed, strain-agnostic antiviral approach. These findings underscore the clinical potential of targeting CDK4- and CDK2-dependent pathways, highlighting GW8510 as a promising candidate for therapeutic development against diverse, real-world HSV-1 infections.

## DISCUSSION

Discovering novel modes of antiviral activity is crucial for designing new drugs that can effectively target drug-resistant viruses. This necessity has been underscored by the recent pandemic, where previously effective drugs have shown minimal efficacy against a new, aggressive virus of the same family. Previously, we hypothesized that the antiviral activity of BX795 stems from the inhibition of EIF4EBP1 hyperphosphorylation (40). Our study, however, also highlighted some shortcomings of BX795, including a narrow therapeutic window. This led to our hypothesis that understanding the mechanism of antiviral action of BX795 can help us design or discover novel drugs that function through an alternative pathway to target viral replication. To understand the mechanism of BX795’s antiviral activity, we performed transcriptomic and proteomic profiling of mock or BX795-treated HCE cells. Our analysis showed that BX795 was a cell cycle modulator with potent CDK2 activity, which can not only block viral DNA replication but also inhibit viral protein translation by inhibiting 4EBP1. Furthermore, our kinase activity assay revealed that CDK4 and CDK6 are also key players in this antiviral mechanism, with CDK4 being the most prominent inhibitor of viral replication. Through connectivity map analysis, we discovered that GW8510 is a functional analog of BX795, which inhibits CDK4 more effectively than CDK2, as determined by a kinase activity assay.

Our findings should also be viewed in the broader context of how herpesviruses co-opt the host cell cycle machinery. Past studies have shown that HSV-1 imposes G1/S checkpoint dysregulation or G1/S arrest, thereby redirecting the DNA replication apparatus toward viral genome amplification (51). This strategy appears conserved across the Herpesviridae: lytic infection by α-, β-, and γ-herpesviruses frequently manipulates the G1/S transition, often through the Rb/E2F axis and upstream CDKs, to optimize the cellular environment for viral DNA synthesis while attenuating host DNA replication (52). Our omics-driven identification of E2F1 as a dominant transcriptional node, coupled with the strong dependence of HSV-1 replication on CDK2 and CDK4, provides direct experimental support for this model in primary human corneal epithelium, a tissue that has been relatively underexplored compared with fibroblasts or transformed cell lines. Recent single-cell work has further suggested that HSV-1 initiates infection across all cell-cycle phases but preferentially establishes productive replication in S/G2 cells, consistent with a requirement for an active DNA synthesis machinery (53). Our data extend these observations by showing that targeted disruption of CDK2/CDK4-driven E2F activity and 4EBP1-dependent translation in corneal cells is sufficient to inhibit this pro-viral cell-cycle state.

Our work also goes in line with a growing body of literature demonstrating that pharmacological CDK inhibitors can function as host-directed antivirals (52). More than two decades ago, Schang and colleagues showed that broad CDK inhibitors suppress replication of HSV-1, HSV-2, and HIV-1, including acyclovir-resistant strains, by targeting cellular rather than viral kinases (54). More recently, the CDK1/2 inhibitor BMS-265246 was reported to potently limit HSV-1 multiplication in several cell types, further validating CDKs as antiviral targets (55). In β-herpesviruses such as human cytomegalovirus (HCMV), both viral and cellular CDKs are being actively explored as drug targets, and FDA-approved CDK4/6 inhibitors, such as abemaciclib have shown robust anti-HCMV activity *in vitro* (55). Our findings extend this paradigm to primary human corneal epithelium and establish GW8510 as a more beneficial functional analog of BX795, which can be used as a CDK-targeting agent that could be optimized for ocular use.

Importantly, our data clarify and refine the mechanism of BX795 in the context of HSV-1 infection. BX795 has been classically described as an ATP-competitive inhibitor of PDK1 and TBK1. Initial antiviral studies in corneal epithelial and other human cell lines attributed its activity to the inhibition of TBK1-dependent pathways and AKT phosphorylation. Subsequent *in vitro* kinase profiling, however, hinted at substantial off-target inhibition of CDK2 at sub-micromolar concentrations. Our integrated multi-omic and biochemical analyzes provide direct evidence that, at the micromolar concentrations required for robust anti-HSV effects in corneal cells, BX795 functions primarily as a pan-CDK inhibitor with particularly high activity against CDK2, CDK4, and CDK6, thereby converging on both cell cycle control and 4EBP1-mediated translational regulation. This mechanistic shift, from a TBK1-centric to a CDK-centric view of BX795, helps reconcile the concentration-dependent divergence between its canonical immunomodulatory properties and its pronounced antiviral activity.

The identification of GW8510 as a functional analog of BX795 via connectivity map analysis is notable for several reasons. GW8510 is a well-characterized CDK2 inhibitor with additional activity against ribonucleotide reductase M2 (RRM2), a key enzyme in dNTP synthesis. Our kinase profiling reveals that GW8510 is particularly effective against CDK4 and CDK2 in corneal epithelial cells. Our functional assays confirm that it blocks RB phosphorylation and preserves nuclear HDAC-chromatin interactions, resulting in potent suppression of HSV-1 replication. The dual targeting of CDK2/CDK4 and RRM2 is mechanistically attractive in the context of a DNA virus that is highly dependent on both S-phase entry and nucleotide availability. From a translational standpoint, GW8510’s antiviral activity at lower molar concentrations and with a more favorable toxicity profile than BX795 in HCEs suggests that rationally designed CDK-focused analogs could retain antiviral potency while widening the therapeutic window.

Our findings also have implications for combination therapy and resistance management in ocular HSV disease. Host-directed CDK inhibition is orthogonal to the mechanism of nucleoside analogs such as acyclovir and ganciclovir, which require viral thymidine kinase for activation. Because CDK inhibitors act on cellular kinases, they are expected to retain efficacy against thymidine kinase–deficient or DNA polymerase–mutant HSV-1 strains that are clinically resistant to standard therapy. Indeed, early work with pharmacological CDK inhibitors demonstrated activity against drug-resistant HSV isolates and synergistic effects with interferon-β, suggesting that CDK modulation can sensitize viruses to innate immune responses.

Our data indicate that BX795 and GW8510 at antiviral concentrations markedly reduce proliferation and deplete S-phase cells *in vitro*, raising important questions about how such agents might impact corneal epithelial turnover, barrier function, and nerve regeneration *in vivo*, especially during active ulceration. Previous topical studies of BX795 on murine corneas reported good tolerability without overt toxicity, but more detailed assessments of epithelial wound healing, stromal keratocyte viability, endothelial integrity, and long-term neurotrophic function will be essential (43). Future work should therefore include *in vivo* dosing studies in models of epithelial keratitis and stromal keratitis, pharmacokinetic profiling of ocular penetration and wash-out, and evaluation of combination regimens with standard antivirals and corticosteroids.

Finally, our multi-omic and CMAP-guided approach exemplifies a generalizable strategy for repurposing and mechanistically deconvoluting small molecules with antiviral potential. By integrating transcriptomic and proteomic signatures of both infection and drug treatment, we were able to (i) pinpoint CDK-E2F-driven cell-cycle reprogramming as a central vulnerability exploited by HSV-1 in corneal epithelium, (ii) redefine the primary antiviral mechanism of BX795 as pan-CDK inhibition impacting both DNA replication and protein translation, and (iii) identify GW8510 as a more CDK-focused analog with favorable antiviral and toxicity profiles. Similar perturbational analyses could be applied to other host-targeted candidates and other herpesviruses, including those where CDK8, CDK9, or viral cyclin–CDK complexes are already recognized as critical, potentially enabling the development of broad-spectrum, resistance-resilient antivirals that can be tailored for topical ocular delivery.

In conclusion, targeting the CDK2 and CDK4 pathways can significantly inhibit HSV-1 replication. The drugs, such as BX795 and its functional analog, GW8510, can enhance the efficacy of the currently available antiviral arsenal and prevent the spread of globally prevalent infection.

## MATERIALS AND METHODS

### Chemicals

All drugs mentioned in this manuscript were purchased from Selleck Chemicals unless mentioned otherwise.

### Cells and viruses

Human corneal epithelial cells (RCB1834 HCE-T) were obtained from Kozaburo Hayashi (National Eye Institute, Bethesda, MD). African green monkey kidney (Vero) cells were provided by P. G. Spear (Northwestern University). Madin-Darby bovine kidney (MDBK) cells were kindly provided by Prof. Richard Longnecker’s laboratory from Northwestern University. HCEs were cultured in minimum essential medium (MEM) (Life Technologies, Carlsbad, CA) with 10% fetal bovine serum (FBS) (Sigma-Aldrich, St. Louis, MO) and 1% penicillin/streptomycin (P/S) (Life Technologies. Vero cells were passaged in Dulbecco’s modified Eagle’s medium (DMEM) supplemented with 10% FBS and P/S.

Unless mentioned otherwise, all infection reporter virus-related experiments were performed using HSV-1 strains 17 GFP and Kos-Dual color ICP0-GFP/gC-RFP. HSV-1 17-GFP (17syn+ background), containing a CMV-GFP expression cassette inserted into the nonessential US5 locus, was originally described by Lilley et al. (56) and HSV-1 KOS strains were kindly provided by Patricia Spear’s laboratory at Northwestern University. This strain has a GFP gene tagged to a cytomegalovirus promoter and produces GFP protein upon successful infection of the virus. Kos-Dual color ICP0-GFP/gC-RFP was kindly provided by Dr. Paul R. Kinchington (University of Pittsburgh). This strain consists of a GFP gene under the control of the ICP0 promoter and an RFP gene under the control of the gC promoter, and thus can differentiate between early and late viral transcription (57). HSV-1 McKrae strain was provided by Prof. Homayon Ghiasi (Cedars-Sinai Medical Center, Los Angeles). ULS and BLS strains of HSV-1 were isolated during our earlier study from ocular material of patients exhibiting various clinical features of HSV infection, including herpetic stomatitis, herpes labialis, and keratoconjunctivitis (50).

### Immunoblotting

Samples were collected and treated with 100 µL of radioimmunoprecipitation assay (RIPA) buffer containing protease and phosphatase inhibitor cocktail (Halt, 78440) for 30 min on ice. Protein extracts (supernatant) were collected by centrifugation at 12,500 × *g* on a bench-top refrigerated (4°C) centrifuge for 15 min. These samples were denatured in NuPAGE LDS Sample Buffer (Invitrogen, NP00007) and β-mercaptoethanol by heating them to 80°C for 10 min. The denatured protein samples were then loaded in equal amounts to 4%–12% SDS-PAGE loading gels, and electrophoresis was performed at a constant speed of 70 V for 3 h. The contents of the gel were transferred to a nitrocellulose membrane using an iBlot two dry transfer system (Thermofisher Scientific, USA). The membrane was blocked (5% nonfat milk in Tris-buffered saline [TBS] and 0.1% Tween 20 [TBST]) for 1 h, followed by incubation of the membrane with the primary antibody overnight at 4°C. The blots were then washed with TBST, followed by incubation at room temperature with the secondary antibody in blocking buffer, before being imaged. All the primary antibodies were used at a dilution of 1:1,000 unless they were for phosphorylated proteins. Secondary IgG antibodies were used at a dilution of 1:10,000. Anti-HSV-1 gB mouse monoclonal antibody (Abcam, 6506) and anti-GAPDH (Proteintech, 10494-1-AP) were used to evaluate the extent of viral infection. All other antibodies referred to in this paper were purchased from Cell Signaling Technologies. Protein bands were visualized using an ImageQuant LAS 4000 imager (GE Healthcare Life Sciences). The density of the bands was quantified using ImageQuant TL image analysis software (version 7). GAPDH was measured as a loading control. Full-sized uncut blots are shown in the supporting files. The list of antibodies used, molecular weights, and their catalog numbers is provided in Table 1.

**TABLE 1:** Antibodies

Antibody	Cat. no.	Company	Mol wt. (kDa)
HSV1-ICP0	ab6513	Abcam, USA	~100–110
HSV1-gB	ab6506	Abcam, USA	~100–111
CDK2	ab101682	Abcam, USA	34
CDK4	A0366	ABclonal, USA	34
RB	A3618	ABclonal, USA	110
Phospho-Rb (Ser608)	#2181	Cell Signaling Technologies, USA	110
E2F-1	#3742	Cell Signaling Technologies, USA	70
Phospho-RB-S780	AP0444	ABclonal, USA	100
Phospho-RB-S807/811	AP0484	ABclonal, USA	120
Phospho-RB-S795	AP0088	ABclonal, USA	106
CDK6	A0705	ABclonal, USA	34
GAPDH	D16H11	Cell Signaling Technologies, USA	37
Thymidine kinase 1 antibody 1	8960S	Cell Signaling Technologies, USA	26
Phospho-4E-BP1 (Thr37/46)	236B4	Cell Signaling Technologies, USA	15 to 20
4EBP1 (53H11)	#9644	Cell Signaling Technologies, USA	15 to 20
HDAC1	A19571	ABclonal, USA	~70
Cell cycle phase determination kit	#17,498	Cell Signaling Technologies, USA	N/A^*a*^

^*a*^ “N/A” indicates not applicable.

### Plaque assay

Infected samples were sonicated using a probe sonication system at 70% amplitude For 30 s in a volume of 500 µL in Opti-MEM. Monolayers of Vero cells were used as a basal cell layer to perform a plaque assay. The samples were serially diluted 10-fold in Opti-MEM prior to being overlaid on Vero cells for 2 h. Infected Vero cells were then washed twice with PBS prior to the addition of DMEM mixed with 5% methylcellulose. The plates were incubated at 37°C and 5% CO_2_ until visible plaques appeared. These samples were then fixed with methanol for 10 min and stained with crystal violet to visualize plaque formation. The plaques were manually counted and input into statistical software for further analysis.

### Quantitative PCR assay

RNA from cells was extracted using the Direct-zol RNA extraction kit (Zymo Research) according to the manufacturer’s protocol. Extracted RNA was quantified using Nanodrop (Thermofisher Scientific, USA) and equilibrated for all samples with RNaase-DNaase-free water (Corning, USA) before they were reverse transcribed into cDNA using High-Capacity cDNA Reverse Transcription Kit (Applied Biosystems, Foster City, CA). Equal amounts of complementary DNA were analyzed via real-time quantitative PCR using Fast SYBR Green Master Mix (Applied Biosystems 4385610) on QuantStudio 7 Flex system (Applied Biosystems). The primers used in this study are shown in Table 2.

**TABLE 2:** Primers

Name	Sequence
CDK11B forward	GCATGCTAGAGTGAAAGAAAAAGAA
CDK11B reverse	CTCCAAGCGGTCCCTTTCTC
CDK11A forward	GAGGATACTTCTGGCGAGCG
CDK11A reverse	GTTAAAACACCCTACGGGGC
p53 forward	AAGTCTAGAGCCACCGTCCA
p53 reverse	CAGTCTGGCTGCCAATCCA
TGF-beta forward	AACCGCACTGTCATTCACCA
TGF-beta reverse	AGCAATGGTAAACCTGAGCCA
CDK4 forward	GCGTGAGGGTCTCCCTTGAT
CDK4 reverse	CCATAGGCACCGACACCAAT
CDK6 forward	TCTGATTACCTGCTCCGCGA
CDK6 reverse	CAGAATCATTGCACCTGAGGG
p21 forward	CACAGGGGAGTTTACGGGAA
p21 reverse	CAGCCTGCGGGTTTTTCTTC
CDC25 forward	CCCTACCTCAGAAGCTGTTGG
CDC25 reverse	TCATCTGGGTCGATGAGCTG
GADD45 forward	AGAGCAGAAGACCGAAAGGATG
GADD45 reverse	TACACCCCGACAGTGATCGT

### Confocal microscopy imaging

Briefly, HCEs were plated in a 96-well plate to perform the experiment. Then, 60% confluent cells were then transfected with either scrambled or CDK2 siRNA for a period of 24 h. The transfected cells were then infected with HSV-1 at a 0.1 MOI for a period of 24 h. At the end of the experiment, the cells were washed in PBS, fixed with 4% paraformaldehyde (PFA; Electron Microscopy Sciences, Hatfield, PA) for 10 min, and then washed again with PBS. A permeabilization and blocking step was performed using 0.01% Triton-X (Fisher Scientific) in 1% bovine serum albumin (BSA; Sigma-Aldrich) for 1 h. Each row of the 96-well plate was incubated overnight at 4°C with a different antibody in 1% BSA. Following incubation, the cells were washed and incubated with an Alexa-fluor-647 conjugated secondary antibody in 1% BSA for 24 h at 4°C. Fluorescent confocal imaging was performed on a LSM 710 confocal microscope (Carl Zeiss) under 20× objectives. A new protocol was developed for this manuscript to image all cells in a 96-well plate by generating a library of x, y, and z locations in Tile mode using Zen Black Software. Once the location of the cells was individually input for each well, the system automatically imaged all the wells, providing an image for every individual well in three channels (DAPI, GFP, and Alexa-fluor 647). The images were saved and named according to the primary antibody used on the 96-well plate.

### Cell cycle analysis

HCEs were incubated in serum-free medium for 6 h to synchronize the cell cycle in all cells. The cells were then treated with BX795 or infected with HSV-1 and collected at various times using Hank’s enzyme-free cell dissociation buffer (Gibco, 13150). Cells were washed once with PBS before being fixed with 4% paraformaldehyde (Electron Microscopy Sciences, USA), followed by permeabilization with 0.01% Triton X-100 for 10 min. The samples were then suspended in PBS containing RNase to digest all RNA present in the cells. The samples were then stained with propidium iodide to stain the DNA present in the cells. Cell suspensions were filtered through a 70-μm mesh, resuspended in PBS, and then analyzed by flow cytometry (BD Accuri C6 Plus). A minimum of 10,000 events was collected for each sample, and cell cycle analysis was performed using FlowJo (version 10).

### Cell proliferation assay

A cell proliferation assay was performed using CytoPainter (Abcam-ab176735) according to the manufacturer’s protocol. Cytopainter powder dye was dissolved in 500 µL of DMSO and stored as 10-µL aliquots at −20°C. HCEs were plated in a 24-well plate format at a seeding density of 10,000 cells/well. The Cytopainter dye was diluted in PBS to 2 µL/mL prior to being added to HCEs in a media-free environment for 30 min in a cell culture incubator. The cells were then washed with PBS, and fresh media containing either 10 µM BX795 or DMSO vehicle control was added to the cells. The cells were incubated for 72 h in the cell culture incubator to allow for proliferation. Cells were detached using Hanks’-based, enzyme-free cell dissociation buffer, washed with PBS through centrifugation at 800 × *g* twice, and then fixed with 4% paraformaldehyde. The cells were then strained through a 70-µm filter before being analyzed using a flow cytometry machine (BD Accuri C6). The fluorescence intensity was gated in the FITC channel to detect cell populations with varied amounts of fluorescence intensity. As a positive control, 10,000 cells freshly stained with Cytopainter dye were also processed to signify the original fluorescence intensity of non-divided cells. Vehicle-treated control samples corresponded to the non-treated group that proliferated during the 72-h incubation period. All the results were processed on FlowJo v10.

### Isolation of primary HCEs

Donated human corneas were gently washed, and the iris from the sclera was removed before placing the epithelium down into 1% dispase solution overnight at 4°C. The next day, using a sterile blunt spatula, cells were dislodged from each of the corneas in PBS. Collected cells were centrifuged and resuspended in 1 mL of 0.5% trypsin for 30 s. Trypsin was neutralized with the help of a 10% FBS solution in PBS, and the cells were centrifuged at 800 × *g* for 5 min. Collected cells were resuspended in keratinocyte media with 1% P/S and 10% FBS. The cells were then allowed to grow in a precoated flask (Gibco Coating Matrix) for 2 weeks. The first two passages of cells were discarded, and only the cells from the third passage were used for the experiments.

### RNA sequencing

Primary human corneal epithelial cells (pHCEs) were used in this experiment. Donated human corneas (Eversight) were gently washed, and the iris from the sclera was removed prior to placing them in 1% dispase solution overnight at 4°C. The next day, using a blunt spatula, cells were dislodged from each of the corneas in PBS. The collected cells were centrifuged and then resuspended in 1 mL of 0.5% Trypsin for 30 s. Trypsin was neutralized with the help of a 10% FBS solution in PBS, and the cells were centrifuged at 800 × *g* for 5 min. Collected cells were resuspended in keratinocyte media with 1% penicillin/streptomycin and 10% FBS. The cells were then allowed to grow in a pre-coated flask (Gibco Coating matrix) for 2 weeks. The first two passages of cells were discarded, and only the cells from the 3rd passage were used for the experiments. pHCEs were either not infected or infected with HSV-1 strain 17 GFP with an MOI of 0.1 and then treated with DMSO or BX795 (10 µM) for a period of 24 h. At the end of 24 h, cells were collected and separated into two equal parts. RNA isolation was performed on one of these parts using Qiagen’s RNA isolation kit using the manufacturer’s protocol. The quality of the RNA was determined using the Life Technologies Qubit instrument prior to sending the samples to LC Sciences. The RNA sequencing was performed by LC Sciences. Briefly, Poly(A) RNA sequencing library was prepared following Illumina’s TruSeq-stranded-mRNA sample preparation protocol. RNA integrity was checked with the Agilent Technologies 2100 Bioanalyzer. Poly(A) tail-containing mRNAs were purified using oligo-dT magnetic beads with two rounds of purification. After purification, poly(A) RNA was fragmented using divalent cation buffer at elevated temperature. The DNA library construction is shown in the following workflow. Quality control analysis and quantification of the sequencing library were performed using an Agilent Technologies 2100 Bioanalyzer High Sensitivity DNA Chip. Paired-end sequencing was performed on Illumina’s NovaSeq 6000 sequencing system.

#### Transcript Assembly

First, in-house Cutadapt and Perl scripts were used to remove reads containing adaptor contamination, low-quality bases, and undetermined bases. The sequence quality was verified using FastQC. Reads were then mapped to the human reference genome (Ensembl release 96) using HISAT2. The mapped reads of each sample were assembled using StringTie. Then, all transcriptomes were merged to reconstruct a comprehensive transcriptome using Perl scripts and gffcompare. After the final transcriptome was generated, StringTie and edgeR were used to estimate the expression levels of all transcripts.

#### Different expression analysis of mRNAs

StringTie was used to perform expression level analysis for mRNAs by calculating FPKM. The differentially expressed mRNAs were selected with log2 (fold change) >1 or log2 (fold change) <−1 and with statistical significance (*P*-value < 0.05) by the R package edgeR.

### Mass spectrometry–based proteomic analysis

Mass spectrometry was performed at the University of San Diego, La Jolla. Whole-cell pellets of primary HCEs, snap-frozen in liquid nitrogen, were sent for proteomic analysis in triplicate. The procedure for the processing of the samples is described elsewhere. All data generated from proteomic analysis were received as a normalized data set in an Excel sheet, which was then loaded into GraphPad Prism software to perform statistical analysis.

### Kinase profiling

The extent of kinase activity inhibition was performed by Reaction Biology. While BX795 was tested in 10-dose IC50 mode with a threefold serial dilution starting at 30 μM, GW8510 was tested only at 10 μM in triplicate. Staurosporine was tested alongside in a 10-dose IC50 mode with a fourfold serial dilution starting at 20 μM, serving as a positive control for all reactions, except for CDK12 (R722C)/cyclin K, CDK12/cyclin K, and CDK13/cyclin K, for which THZ531 was used. The reactions tested were CDK1/cyclin A, CDK1/cyclin B, CDK1/cyclin E, CDK12 (R722C)/cyclin K, CDK12/cyclin K, CDK13/cyclin K, CDK14/cyclin Y (PFTK1), CDK16/cyclin Y (PCTAIRE), CDK17/cyclin Y (PCTK2), CDK18/cyclin Y (PCTK3), CDK19/cyclin C, CDK2/cyclin A, CDK2/cyclin A1, CDK2/CYCLIN E, CDK2/cyclin E2, CDK2/cyclin O, CDK3/cyclin E, CDK3/cyclin E2, CDK4/cyclin D1, CDK4/cyclin D2, CDK4/cyclin D3, CDK5/P25, CDK5/p35, CDK6/cyclin D1, CDK6/cyclin D2, CDK6/cyclin D3, CDK7/cyclin H, CDK8/cyclin C, CDK9/cyclin K, CDK9/cyclin T1, and CDK9/cyclin T2. All reactions were performed at a concentration of 10 μM ATP.

### Protein docking studies

Experimentally determined three-dimensional structure of CDK1, CDK2, CDK4, CDK5, CDK6, CDK8, and CDK9 was retrieved from RCSB PDB (https://www.rcsb.org/). All the ligands were then converted into .pdbqt format using Autodock Tools (21). Using a text editor, water, ligands, cofactors, and ions were removed from the protein PDB files. This was followed by the preparation of proteins for docking studies using Autodock Tools. Briefly, polar hydrogens were added to the protein, and a grid box with a spacing of 1 Å was set up to encompass the ATP binding region. Ultimately, each protein was saved in .pdbqt format. Ligands BX-795 and AZD5363 were downloaded from PubChem. 3D cleaning, adding explicit hydrogens, and structural checks were performed using MarvinSketch (Marvin version# 19.18.0). Docking studies were performed using Autodock Vina software with an exhaustiveness level of 40 on all proteins, one at a time, using ligands BX-795 and a suitable positive control. Visualization of non-bonding interaction between protein-ligand complexes was done using Discovery Studio (BIOVIA, D. S. Discovery Studio Visualizer, v19. 1.0. 1828 (2019). San Diego: Dassault Systèmes).

### siRNA transfection of HCE cells

HCE cells were transfected with small interfering RNA (siRNA) to achieve transient knockdown of target genes. Cells were seeded at ~50%–60% confluence in DMEM 24 h before transfection. Gene-specific siRNA and non-targeting control siRNA at a final concentration of 10 nM were mixed with RNAiMAX in Opti-MEM reduced-serum medium according to the manufacturer’s protocol. The siRNA-lipid complexes were added dropwise to the cells and incubated for 8 h. The cells were incubated for an additional 48 h under standard culture conditions. Knockdown efficiency was assessed by qRT-PCR and Western blotting. Cell viability was monitored to confirm that the transfection procedure did not induce cytotoxicity. All experiments were performed in parallel with a non-targeting siRNA control.

### Cell viability assay (MTT)

The *in vitro* cytotoxicity of GW8510 and DMSO was evaluated using a standard MTT assay on HCE cells. Briefly, a 1 × 10^4^ monolayer of HCEs per well in a 96-well plate was treated with different concentrations of GW8510. Twenty-four hours post-treatment, the assay was terminated. MTT reagent was added to each well at a concentration of 0.5 mg/mL, and the plate was incubated for an additional 4 h. The intensity of the color developed was then analyzed using a Tecan GENios Pro microplate reader at 562 nm. Experiments were conducted using three biological replicates.

### Statistical analysis

GraphPad Prism software (version 9.0) was used for statistical analysis of the data within each group. *P* < 0.05 was considered a significant difference among the compared groups. One-way ANOVA was used to determine significance for all confocal image fluorescence intensity analyzes, viral plaque counts, and disease score analysis. *P*-values represented as *, **, ***, and **** correspond to values <0.05, <0.01, <0.001, and <0.0001, respectively. A list of differentially expressed genes and proteins, along with normalized FPKM values, *P*-values, and false discovery rates, was provided by the institution to which the analysis was outsourced.

## SUPPLEMENTAL MATERIAL

Supplemental figures mbio.03741-25-s0001.docx Fig. S1 to S4.

ASM does not own the copyrights to Supplemental Material that may be linked to, or accessed through, an article. The authors have granted ASM a non-exclusive, world-wide license to publish the Supplemental Material files. Please contact the corresponding author directly for reuse.
