# Supplementary material for: A CDK-4EBP1 signaling axis drives HSV-1 replication and underscores a druggable pathway for potent antiviral intervention
Source: mBio. 2026 Jan 23;17(2):e03741-25. doi: 10.1128/mbio.03741-25 (PMC12892985; doi:10.1128/mbio.03741-25)

**A CDK-4EBP1 Signaling Axis Drives HSV-1 Replication and Underscores a Druggable Pathway for Potent Antiviral Intervention**

Krishnaraju Madavaraju^1^*, Tejabhiram Yadavalli^1^*, Sudhanshu Kumar Singh^1^, Chandrashekhar D. Patil^1^, Hemant Borase^1^, Deepak Shukla^1,2#^

^1^ Department of Ophthalmology and Visual Science, University of Illinois Chicago, Chicago, IL, USA

^2^Department of Microbiology and Immunology, College of Medicine, University of Illinois Chicago, Chicago, Illinois, United States.

*These authors contributed equally to this work

[^#^dshukla@uic.edu](mailto:#dshukla@uic.edu), corresponding author

**Supplementary Figure Legends**

**Supplementary Figure 1.**

CDK2 knockdown suppresses 4E-BP1 hyperphosphorylation. (A) Confocal immunofluorescence analysis of the 4E-BP1 pathway in non-infected HCE cells transfected with CDK2 or control siRNA. Staining for phosphorylated 4E-BP1 (RED) shows a greatly reduced signal in CDK2 knockdown cells relative to controls, indicating loss of 4E-BP1 hyperphosphorylation. Phospho-p70 S6 kinase (p-P70S6K, red) is similarly diminished upon CDK2 knockdown, consistent with impaired mTORC1 downstream signaling. In contrast, phosphorylated Akt (p-Akt) levels remain unchanged in CDK2-silenced cells compared to control, demonstrating that CDK2 inhibition suppresses 4E-BP1 phosphorylation independently of upstream Akt. Images are representative of three independent experiments. These data confirm that CDK2 depletion significantly impairs HSV-1 infection and disrupts the host's 4E-BP1 (eukaryotic initiation factor 4E-binding protein 1) phosphorylation status. (B) Quantitative analysis of the mean fluorescence intensity of the targets shown in panel A. siCDK2 was used at a concentration of 10 nM. The data represent three experiments. Statistical significance was determined by one-way ANOVA: non-significant, *P < 0.05, **P < 0.01, ***P < 0.001, ****P < 0.0001.

**Supplementary Figure 2.**

BX795 induces the downregulation of CDK1/Cyclin B and stably binds to CDK2. (A) Time-course RT-qPCR analysis of cell-cycle regulators in HCE cells treated with BX795 (10 μM) or DMSO vehicle. Transcript levels of CDK1 and Cyclin B (Cyclin B1) are significantly reduced by 8 hours post-treatment in BX795-treated cells compared to control (P<0.05 vs. DMSO at 8 h; n=3 independent samples), indicating rapid downregulation of G₂/M cell cycle drivers. (B) The number of hydrogen bonds between BX795 and CDK2 remains consistent over time, with no significant loss of interactions, further supporting the stability of the binding. (C) The radius of gyration of the CDK2–BX795 complex remains unchanged during the simulation, indicating that the overall protein structure is stable and not unfolding upon ligand binding. (D) The solvent-accessible surface area (SASA) of the BX795 binding interface on CDK2 also remains constant, with no exposure of buried surfaces, consistent with a stably maintained ligand–protein complex. These in silico results support that BX795 directly engages CDK2 in a stable manner, corroborating the experimental findings that BX795 treatment functionally inhibits CDK2 activity in cells. (E) Molecular dynamics simulation of BX795 bound to CDK2 shows a stable interaction over 50 ns. The root-mean-square deviation (RMSD) of BX795 in the CDK2 active site remains low throughout the simulation, indicating minimal drift from the initial docking pose.

**Supplementary Figure 3.**

BX795 targets multiple CDKs and activates antiviral response pathways. (A) In silico docking analysis of BX795 with various cyclin–CDK complexes. Predicted binding affinities (docking scores) indicate that BX795 binds strongly to several cyclin-dependent kinases, with the highest affinity for CDK4 and CDK6 complexes, in addition to CDK2. (B) Heatmap of the proteomic changes induced by BX795 in HCE cells. Each point represents a protein. significantly upregulated (red) and downregulated (blue) proteins in BX795-treated cells (10 μM, 24 h) versus vehicle-treated cells are highlighted (threshold criteria: P<0.05, fold-change ≥1.5). Approximately 600 proteins are differentially expressed in response to BX795 treatment, indicating a broad range of cellular responses. (C) Gene Ontology (GO) enrichment analysis of genes upregulated by BX795 treatment. Top enriched biological processes include “Defense against virus,” “kinase regulatory activity,” and “DNA/RNA metabolic process,” indicating an antiviral and cell-cycle regulatory transcriptional response (P<0.05, hypergeometric test). (D) GO enrichment analysis of the upregulated proteins in BX795-treated cells shows overlapping themes of antiviral defense and cell cycle regulation. Together, these unbiased transcriptomic and proteomic analyses demonstrate that BX795 triggers antiviral state pathways and broadly impacts cell cycle regulators.

**Supplementary Figure 4**

GW8510 is a potent CDK4/2 inhibitor with broad anti-HSV activity. (A) Kinase assay demonstrating BX795’s activity against CDK2 at low concentration. At 0.1 μM BX795, more than 90% of CDK2 kinase activity is inhibited, consistent with prior reports and confirming that BX795 can act as a CDK2 inhibitor at sub-micromolar concentrations.


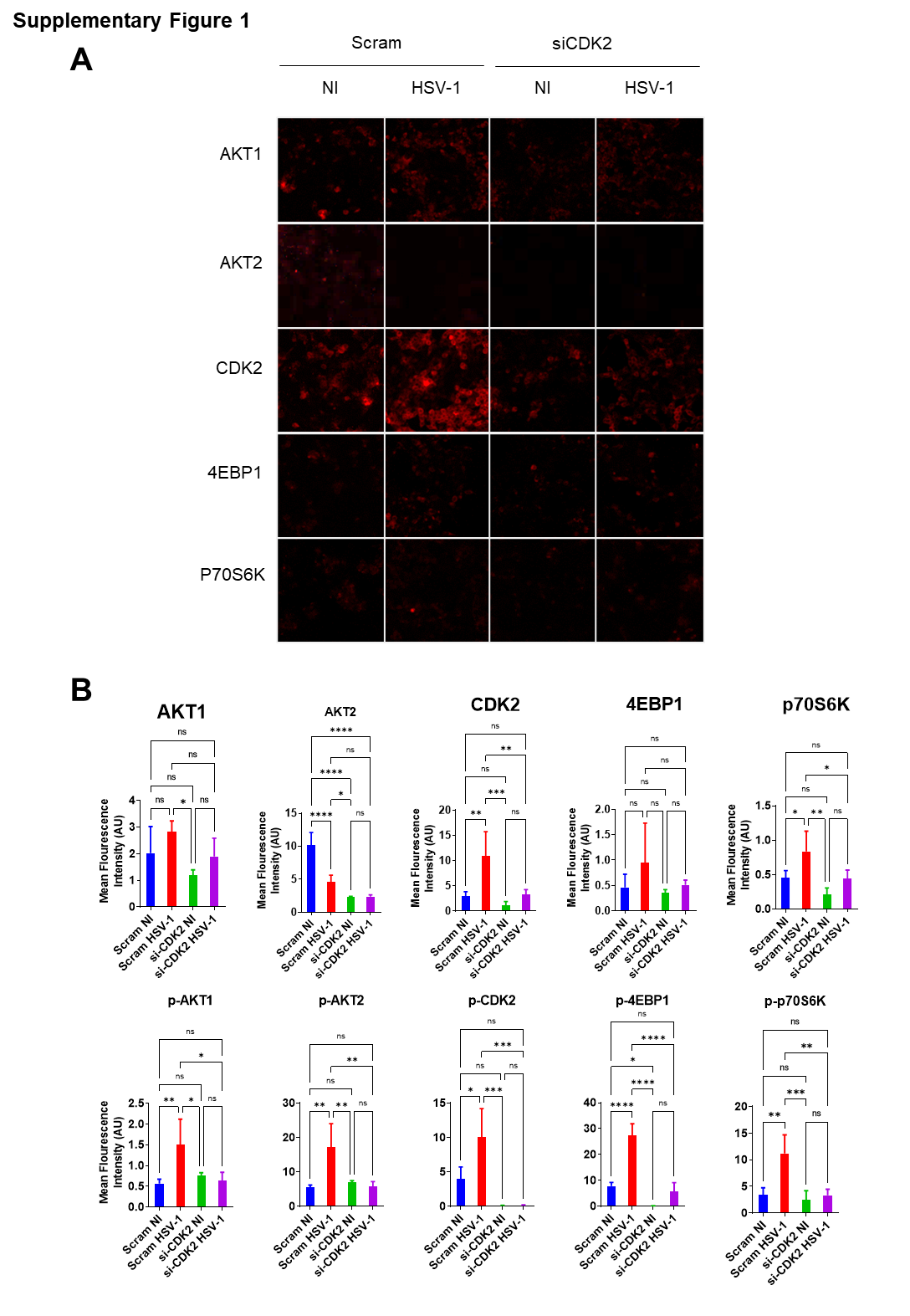


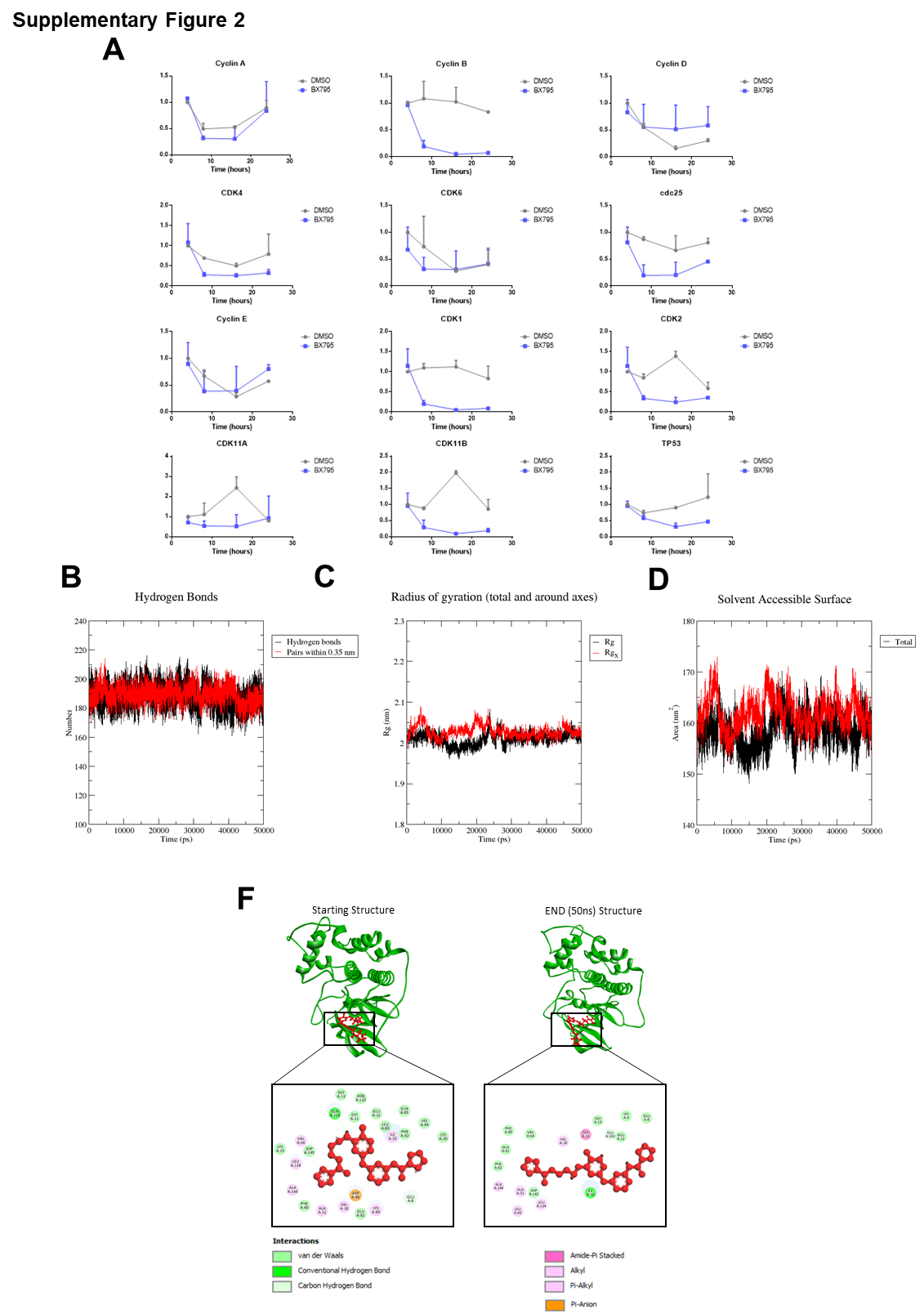


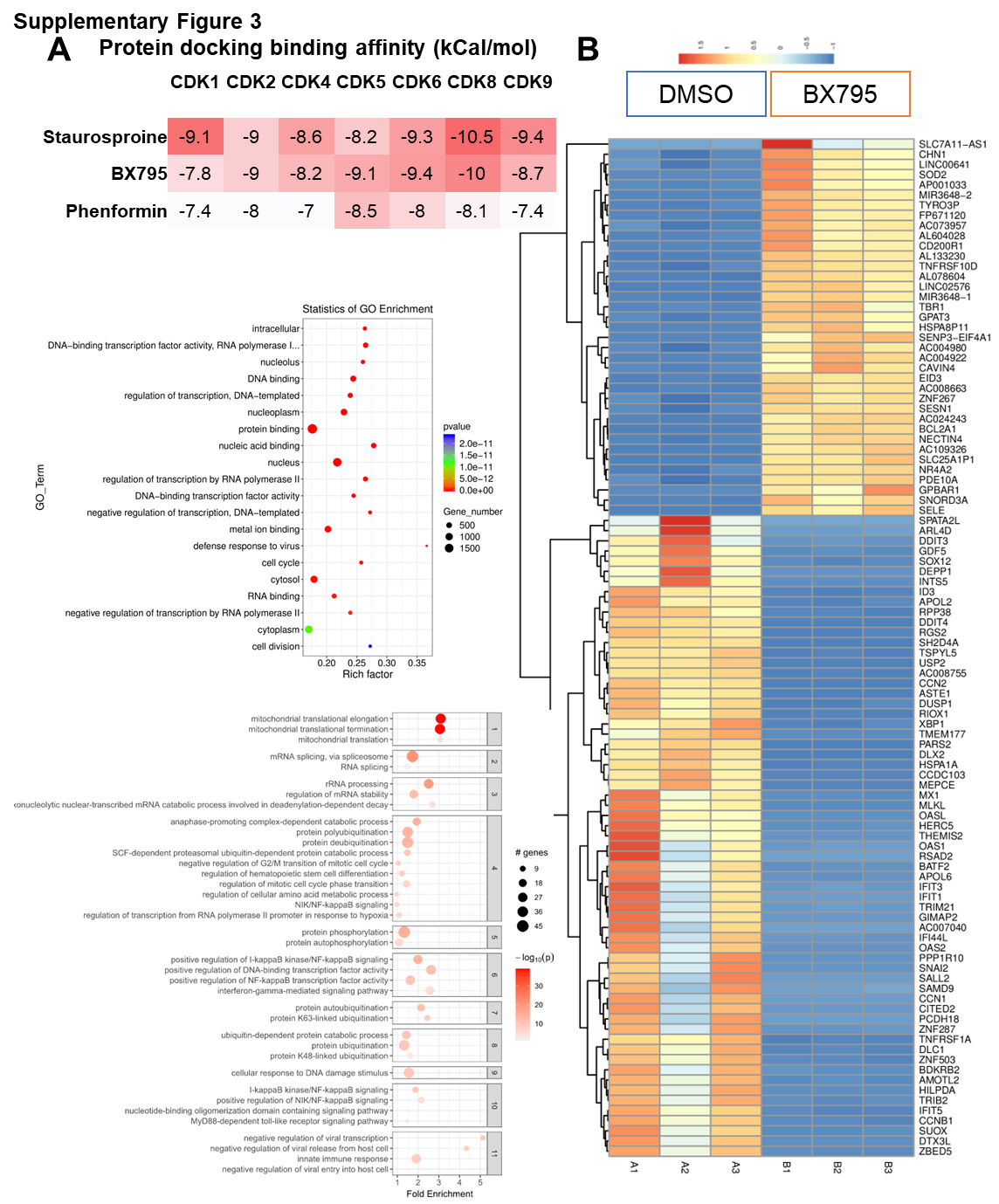


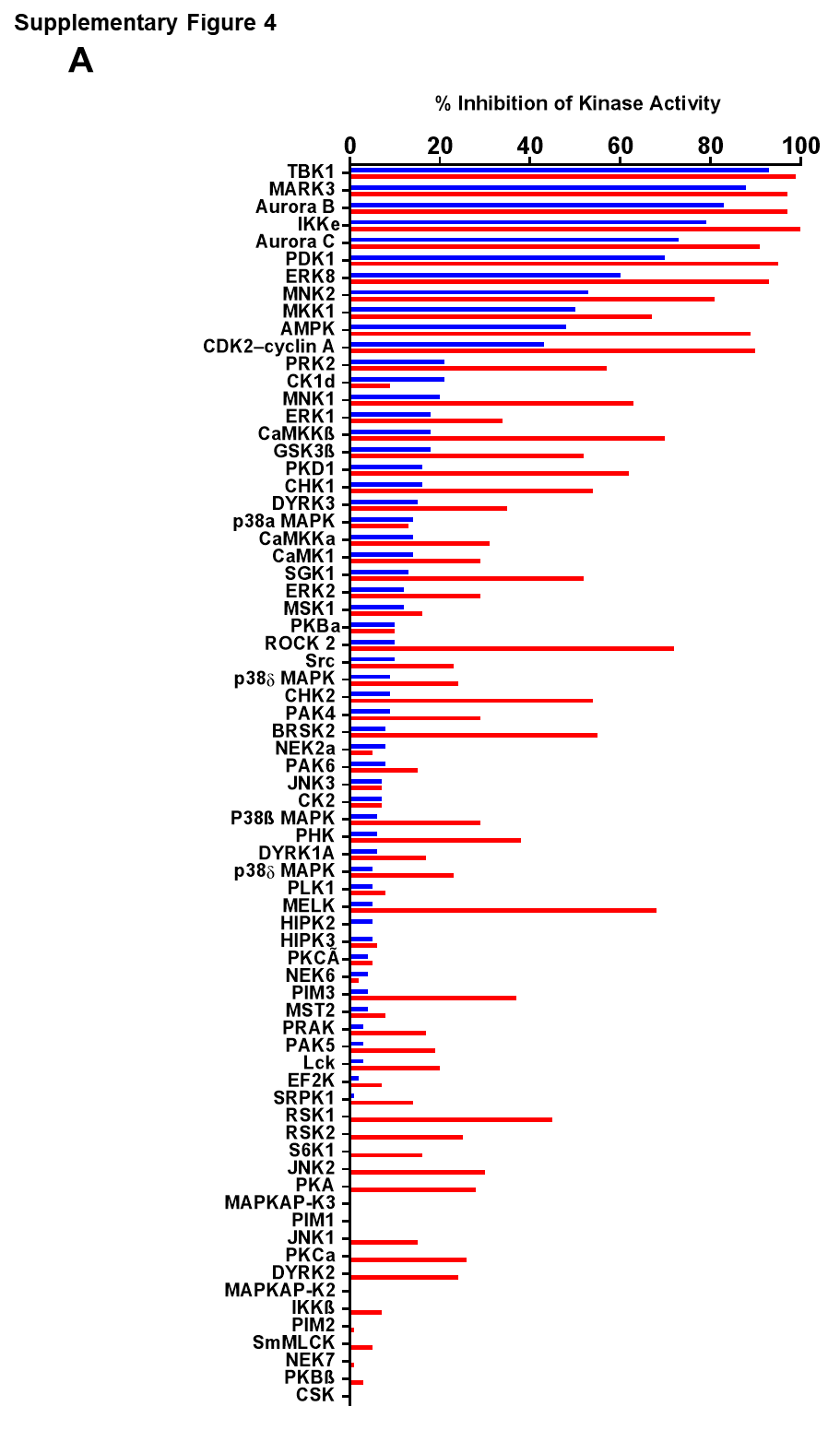

Supplement: Supplemental figures — Fig. S1 to S4. [file mbio.03741-25-s0001.docx]
